# The Hospital Treatment of Epidemic Diarrhœa

**Published:** 1919-07-12

**Authors:** 


### The Hospital Treatment of Epidemic Diarrhœa.

Under the Maternity and Child Welfare Act, 1918, local authorities are empowered to provide hospital treatment for children under the age of five; and specific reference is made to such diseases as epidemic diarrhœa and ophthalmia neonatorum. The provision of institutional treatment for epidemic diarrhœa is a wise procedure, and will undoubtedly result in the saving of many lives, as there is no disease of childhood which requires more sustained and careful effort with regard to nursing and dieting. In the small and crowded homes of the industrial classes efficient nursing and treatment are difficult and frequently impossible to attain. The necessary accommodation for the treatment of epidemic diarrhœa may be provided in general hospitals, children's hospitals, or in cots connected with maternity and child welfare centres. Wherever such provision is made efficient nursing is essential. The admission of cases of epidemic diarrhœa to general hospitals will make a call for an increased nursing staff, as the young children and infants suffering from this disease require constant care and attention both day and night; but no great difficulty should be experienced in making satisfactory and working arrangements between local authorities and the governing bodies of general hospitals. Such arrangements should include accommodation for the admission of mothers of young infants. There may be considerable doubt in certain districts as to the extent to which beds may be available; but by wise foresight on the part of the hospital authorities it should be comparatively easy to give first call on certain beds during the summer months to urgent cases of epidemic diarrhœa.

